# Composite Memristors by Nanoscale Modification of Hf/Ta Anodic Oxides

**DOI:** 10.1021/acs.jpclett.1c02346

**Published:** 2021-09-09

**Authors:** Ivana Zrinski, Alexey Minenkov, Cezarina Cela Mardare, Achim Walter Hassel, Andrei Ionut Mardare

**Affiliations:** † Institute of Chemical Technology of Inorganic Materials, Johannes Kepler University Linz, Altenberger Str. 69, 4040 Linz, Austria; ‡ Christian Doppler Laboratory for Nanoscale Phase Transformations, Center for Surface and Nanoanalytics, Johannes Kepler University Linz, Altenberger Str. 69, 4040 Linz, Austria; § Danube Private University, Steiner Landstrasse 124, 3500 Krems-Stein, Austria

## Abstract

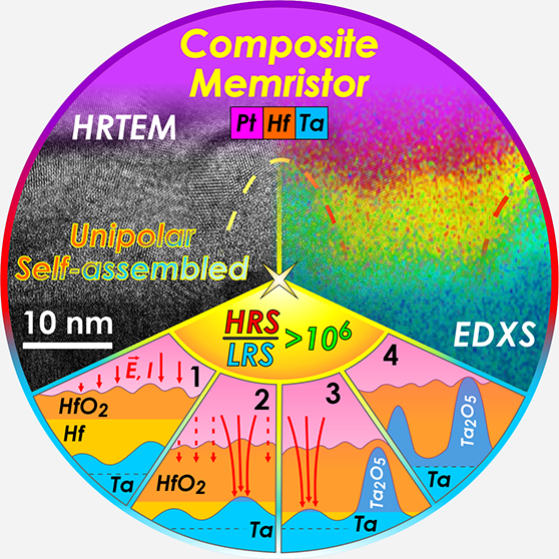

Composite memristors based on anodic oxidation of Hf superimposed on Ta thin films are studied. A layered structure is obtained by successive sputtering of Ta and Hf thin films. The deposition geometry ensured components’ thickness gradient profiles (wedges) aligned in opposite directions. Anodization in citrate buffer electrolyte leads to a nanoscale columnar structuring of Ta_2_O_5_ in HfO_2_ due to the higher electrical resistance of the latter. Following the less resistive path, the ionic current forces Ta oxide to locally grow toward the electrolyte interface according to the Rayleigh–Taylor principle. The obtained composite oxide memristive properties are studied as a function of the Hf/Ta thickness ratio. One pronounced zone prominent for memristive applications is found for ratios between 4 and 5. Here, unipolar and bipolar memristors are found, with remarkable endurance and retention capabilities. This is discussed in the frame of conductive filament formation preferentially along the interfaces between oxides.

Various oxides and their modifications are in scientific focus for memristors fabrication, which are directly applied in nonvolatile memories.^1,2^ Such devices exceed most limits of conventional memory technology.^3^ Memristors are often recognized as resistive random access memories (ReRAMs),^4^ in which the data storage is based on the resistance state change.^3,5^ Their applications extend to logic circuits^6,7^ and units,^8^ neuromorphic systems,^9,10^ sensors,^11,12^ and photodetection.^13^ The switching performance, from a high resistance state (HRS) to a low resistance state (LRS), depends on the selection of electrodes and active/oxide layers. This will define the conductive pathways formation, which is mediated by oxygen vacancies and/or cations and their field-activated movement inside the oxide.^14^

Oxides of valve metals have shown remarkable performances as memristive elements.^15−18^ Studies on Hf- and Ta-based memristors reported excellent electrical and memory properties, such as multilevel switching, high endurance, and data retention.^16,17^ The deposition of oxide layers is commonly done by atomic layer deposition^19,20^ or sputtering.^21^ Alternatively, the electrochemical anodization process is a faster, less complex, and inexpensive method, with precise composition and oxide thickness control through electrochemical parameters.^22,23^

It was confirmed that the performance of Hf or Ta anodic memristors can be improved by carefully selecting the anodization electrolyte or other electrochemical parameters.^16,24^ These play a crucial role in conductive filaments (CFs) positioning and sizing. Such an approach may facilitate defect-engineered memristors fabrication,^18^ which is a major motivation for investigating devices based on mixed oxides formed in different electrolytes. The mixture of HfO_2_ and Ta_2_O_5_ is already recognized as a high-*k* gate dielectric used in field-effect transistors.^25,26^

The aim of this work is to study the behavior of anodic memristors based on Hf superimposed on Ta layers anodized in citrate buffer (CB). *In situ* oxide nanostructuring is reported for several superimposed valve metals, such as Nb/Ta, Nb/Al, or Ta/Al.^27−31^ Their anodization leads to nanoscale oxide columns (or “fingers”) formation, when a metal producing a more resistive oxide is superimposed on a metal producing a less resistive one. This phenomenon is recognized as an electrical version of the Rayleigh–Taylor effect^28,32^ and results from the ionic current preferring the less resistive paths, enhancing the growth of correspondent oxide. Oxide resistivities and structures, transport numbers, and Pilling–Bedworth ratios were all considered as determining factors for the anodization process of such superimposed systems.^28^ In the current work, anodization of the Hf/Ta system leads to the Rayleigh–Taylor effect since HfO_2_ is the more resistive oxide.^33^ The boundary between Hf and Ta oxides may influence the conductive pathways required for the memristive effect, thus being most relevant for fabrication of highly stable and forming-free memristors.

A sputtering system (Mantis Deposition, United Kingdom) was used for sequential deposition of Hf and Ta. General details regarding the thin film deposition can be found elsewhere.^16,24^ In Figure 1, the experimental approach for the sample preparation is presented. For obtaining the desired sample configuration, first, a 300 nm Ta film was uniformly deposited on thermally oxidized Si wafers (100 mm in diameter) by rotating the substrate during deposition with a constant speed of 5 rpm. The uniform Ta plays the role of a bottom electrode in the memristor structure. Then, a thin Ta film was deposited while the substrate position was fixed. In this way, a Ta wedge with thickness gradually varying from 6 to 2 nm was obtained. Afterward, in identical conditions without breaking the vacuum, a Hf film was deposited by using the opposite gun. Thus, a complementary Hf wedge with thickness varying from 3 to 11 nm grew superimposed on the Ta wedge. The different gradients for Hf and Ta were defined aiming for a thickness of the final oxide layer below 20 nm, as recommended for memristor formation.^16,34^

**Figure 1:**
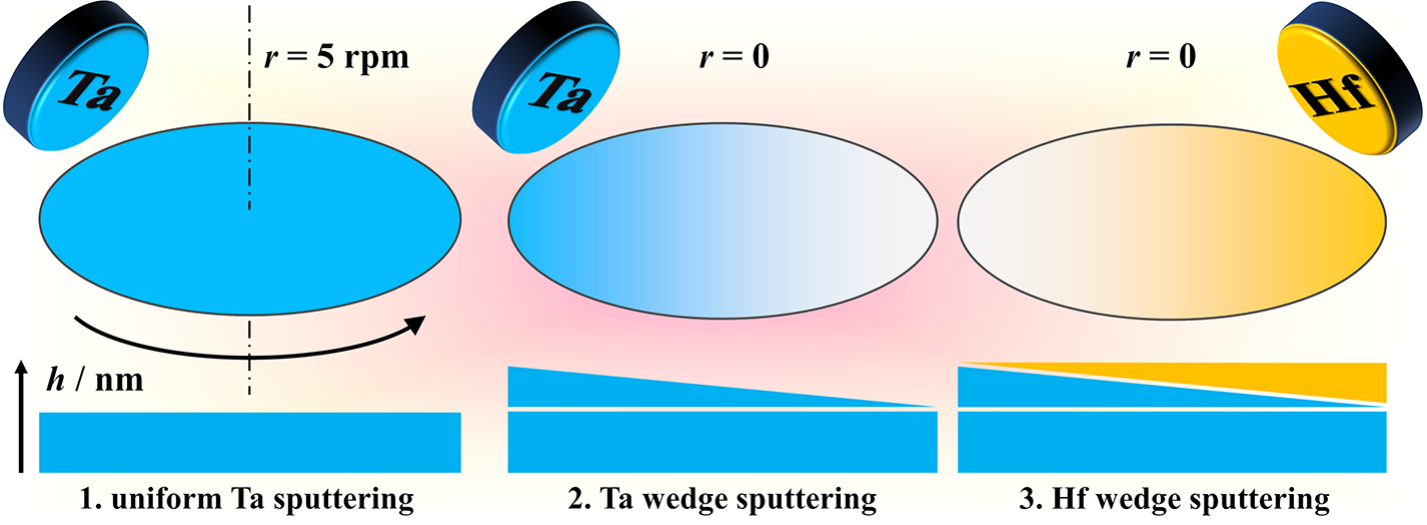
Fabrication steps involved in the deposition of the Hf/Ta system, starting with a uniform Ta bottom electrode and followed by the successive deposition of Ta and Hf thickness gradient thin films.

The Hf–Ta layers were electrochemically oxidized by using potentiodynamic anodization up to 7 V (at a scan rate of 100 mV s^–1^) with a CompactStat potentiostat (Ivium Technologies, The Netherlands). Setup details were recently reported.^24^ The chosen fabrication conditions were optimal for achieving the best memristive performance.^16,34,24^ Following the standard recipes,^35^ 0.1 M citrate buffer (CB), pH = 6.0, was prepared and used for the anodization in ambient conditions. Following the anodic oxide growth, memristors were obtained by sputtering Pt top electrodes (200 μm in diameter) through a shadow mask foil (Mecachimique, France) across the surface of the entire wafer. They were electrically contacted by using a W needle. More fabrication and testing details can be found elsewhere.^16,24^

Memristive effects in the Hf–Ta system were investigated along the wedges by performing typical *I–U* sweeps. The voltage was biased up to 2 V against the bottom electrode with current compliances up to 20 mA, while the top electrode remained grounded. All memristive devices were grouped according to their electrical characteristics, as exemplified in Figure 2. Devices without substantial memristive effects were found either in Ta- or Hf-enriched regions, which are denominated as zones I and III. Memristive effects were found only in a middle zone II, where the parent metals were deposited with a Hf/Ta thickness ratio of ∼4.5. Zone II is rather narrow, its limits being defined by Hf/Ta ratios ranging from 3.9 to 5.1. Outside these thresholds, zones I and III were defined. One 5 × 5 electrode cluster was deemed representative for each zone. Thus, peculiar Hf/Ta ratios with prominent characteristics can be identified for further applications.

**Figure 2:**
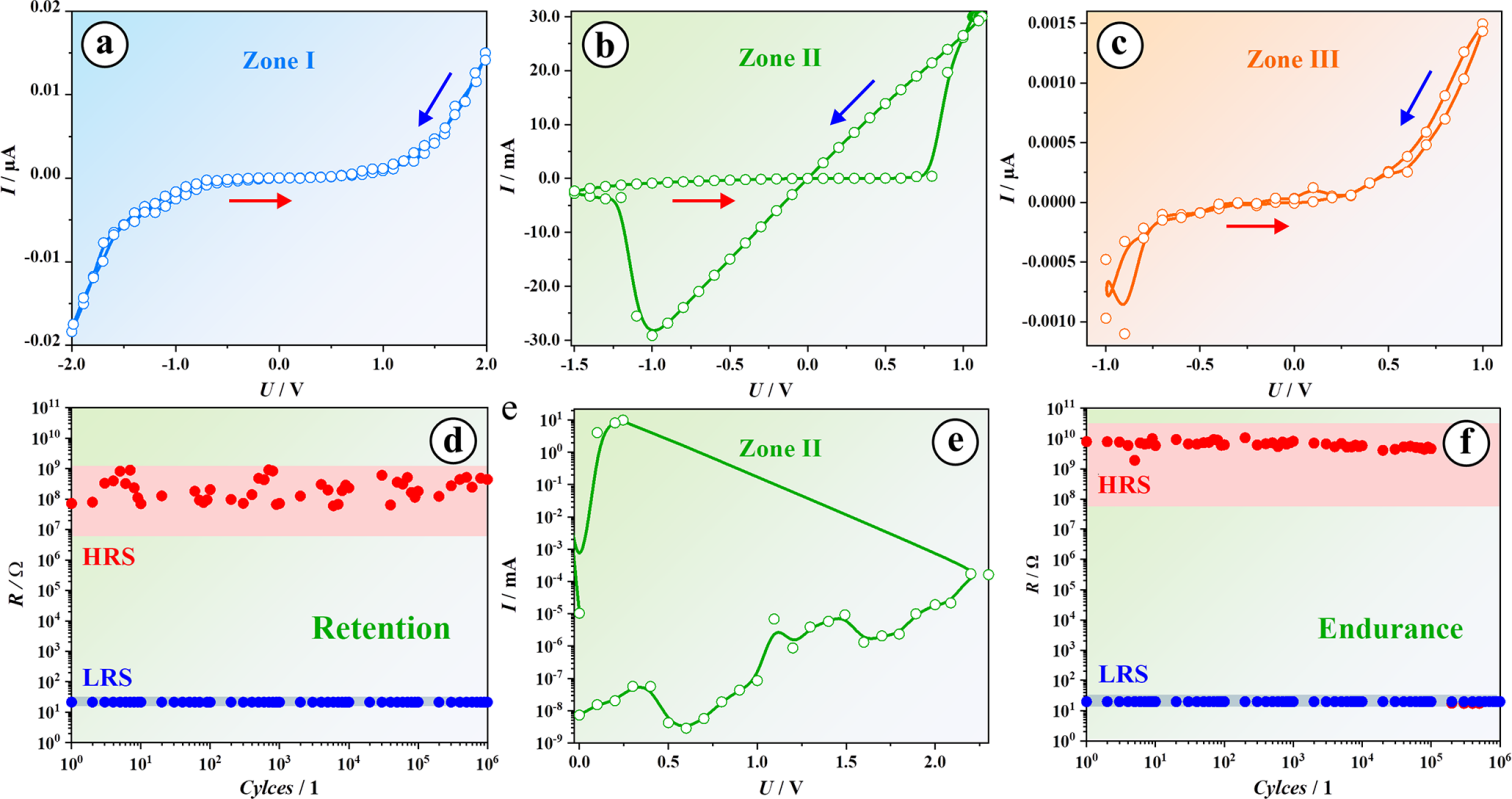
Representative *I*–*U* curves of composite Hf/Ta oxide memristors in three defined zones (a–c, e) and exemplified retention (d) and endurance (f) testing in zone II.

Figure 2a shows the performance of devices fabricated in the Ta-rich zone I. Generally, no hysteretic curve was observed in this zone while performing *I–U* sweeps under controlled voltage and current which never exceeded ±2 V and 500 μA, respectively. Higher voltage ranges resulted in irreversible switching or device breakdown. The resistance values were in the range of GΩ with undistinguishable HRS and LRS. This suggests that no memristive effect was stabilized following the electroforming process by biasing in the range of 4 V. A very similar switching trend was observed in zone III (Figure 2c). In contrast, zone II appears to be a promising region facilitating the fabrication of both bipolar (Figure 2b) and unipolar (Figure 2e) memristors. The resistance state ratio in this zone was in the range of 10^7^, which is a remarkable improvement of devices based on pure Hf or Ta.^16,24,34^ Devices here were initially unipolar and forming free, eventually switching bipolarly after several hundred cycles. Hence, unipolar devices can be tuned to the bipolar mode by a writing procedure resulting in improved endurance, which was until now recognized as very short for unipolar devices.^36^

The specific performance of zone II memristors, showing mixed bipolar/unipolar switching, may be directly linked to its morphological characteristics. To reach this conclusion, it is crucial to consider the reported differences between unipolar and bipolar switching mechanisms. The mechanism of unipolar switching is based on mass transfer assisted by Joule heating that leads to CFs formation or rupture due to thermophoresis and O anion diffusion inside the oxide under field control.^37^ Bipolar switching refers to nanoionic transport during the redox process induced by external electric fields. It was also reported that nanoisland structured CFs can be found in unipolar devices, more likely than continuous filaments creating metallic contacts between electrodes.^38^ Mixed bipolar/unipolar memristive switching was also previously communicated.^38−40^ A mixed behavior is based on a combination of both mechanisms depending on the current compliance. Because it is less likely that relevant Joule heating would be driven at lower current compliances, bipolar switching may take place due to the O vacancies movement. In contrast, when applying higher current compliances, excessive temperatures may be generated and devices would be switched unipolarly.^38^ This may not be in agreement with the results of the current work, taking into account that a current limitation of up to 30 mA was applied during bipolar and unipolar switching of the devices fabricated in zone II (Figures 2b and 2e). This could support only the bipolar behavior of devices in zones I and III (Figures 2a and 2c), where very low current compliances were kept. Nevertheless, it may be already suggested that the mixed behavior observed in composite oxides from zone II is directly related to the expected Rayleigh–Taylor oxide nanostructuring within the active memristive layer and will be discussed further. The fact that oxide nanoislands may form during the anodization process may clarify why all unipolar memristors were forming free. Thus, it may be also inferred that forming-free memristors will show unipolar behavior once the nanoisland regions are more abundant compared to the oxide “fingers” formed or once the CFs are irreversibly oxidized. However, since all unipolar devices will eventually turn into bipolar ones, it may be concluded that the oxide “fingers” play a crucial role in the overall switching behavior. The position and size of CFs could be predefined by these nanostructures. Along their boundaries, O vacancies and metal cations move easier between cathode and anode interfaces to reduce or oxidize CFs. To activate this “fingers” support for CFs in unipolar devices, a pretreatment in the form of consecutive cycling is necessary. Even though this does not simplify the operating mode of the memristors, it still allows controllable switching.

As the next part of memristive investigation, endurance and retention tests were performed. Retention was tested by repeatedly reading the resistance value for a given memristive state by biasing memristors at their switching voltages (*U*_set_ and *U*_reset_), while endurance testing refers to successful cyclic memristive switching at *U*_set_ and *U*_reset_. During endurance and retention testing electrodes were connected in the same manner, and the resistance was read by applying 0.01 V. The frequency of switching between LRS and HRS during the endurance cycling was 260 Hz. As already observed by recording *I–U* sweeps, memristors from zones I and III did not show any reproducible or meaningful endurance/retention data. Thus, only the results for memristors fabricated in zone II are presented in Figures 2d and 2f. Cycle to cycle variability intervals for HRS and LRS are also shown in the figures by red and blue confidence bands, which were empirically extracted from all tests (up to 25 memristors which were successfully switched). For both retention and endurance measurements, LRS values were extremely stable and low (≈20 Ω), showing a minimum cycle to cycle variability for at least 10^6^ cycles. The HRS values showed higher instabilities, varying from 10^7^ to 10^9^ Ω during retention and from 10^8^ to 10^10^ Ω during endurance tests. Resistance states ratio reached 10^8^, which is a drastic improvement when compared to Hf or Ta anodic memristors.^16,17,34^ However, the memristors failed after 10^5^ cycles during consecutive writing, which was previously explained by the sudden diffusion of O vacancies into CFs increasing the conductance of HRS.^41^

Immediately after testing, memristors representative for each zone were analyzed by HRTEM, and the results are summarized in Figure 3. Detailed experimental information was recently reported.^24^ Apart from high-resolution imaging, STEM EDX maps were constructed to localize Hf and Ta species within the composite anodic oxides layer. As intended from the sample preparation step, for all zones the thickness of the composite Hf/Ta oxide is around 15 nm. In all cases the Pt top electrode and composite oxide regions are well-distinguishable by following the Pt and O maps, respectively. In each zone the Rayleigh–Taylor effect can be observed by the Ta oxide columnar growth toward the Pt top electrode. However, its intensity strongly depends on the Hf/Ta ratio. Zone I shows mainly an amorphous oxide characteristic to Ta_2_O_5_,^24^ while a relatively thin polycrystalline Hf-based layer, continuously separating Ta and Pt, is readily visible in the correspondent HRTEM image and EDX map (Figure 3). In contrast, the predominantly polycrystalline structure is specific to the zone III oxide due to the high amount of HfO_2_.^16^ This may also explain the unstable memristive effect of zones I and III, in which HfO_2_ is believed to play the role of a barrier blocking CFs formation through the whole oxide later. Zone II shows a mixture of amorphous and crystalline oxide regions, with visible amorphous Ta_2_O_5_ “fingers” delimited by crystalline HfO_2_ areas, as roughly indicated in the HRTEM image by white dashed lines using EDX elemental map as a reference (Figure 3). In the entire Hf/Ta system, Ta_2_O_5_, being less resistive (and less dense), grows faster toward the electrolyte interface, while HfO_2_ spreads to the metal–oxide interface. This is stepwise described in the suggested model (Figure 4a). First, the Hf/Ta structure formed by films with certain surface roughness is exposed to the electrolyte and the electrical field is applied. Being on top, Hf converts into oxide in a rather uniform field. As soon as the anodization front reaches Ta, Ta_2_O_5_ is formed, and this becomes a preferential path for the current flow. As a result, a column of Ta_2_O_5_ grows much faster compared to the surrounding HfO_2_. The process repeats at different locations until finally both layers are completely transformed into anodic oxides (Figure 4a).

**Figure 3:**
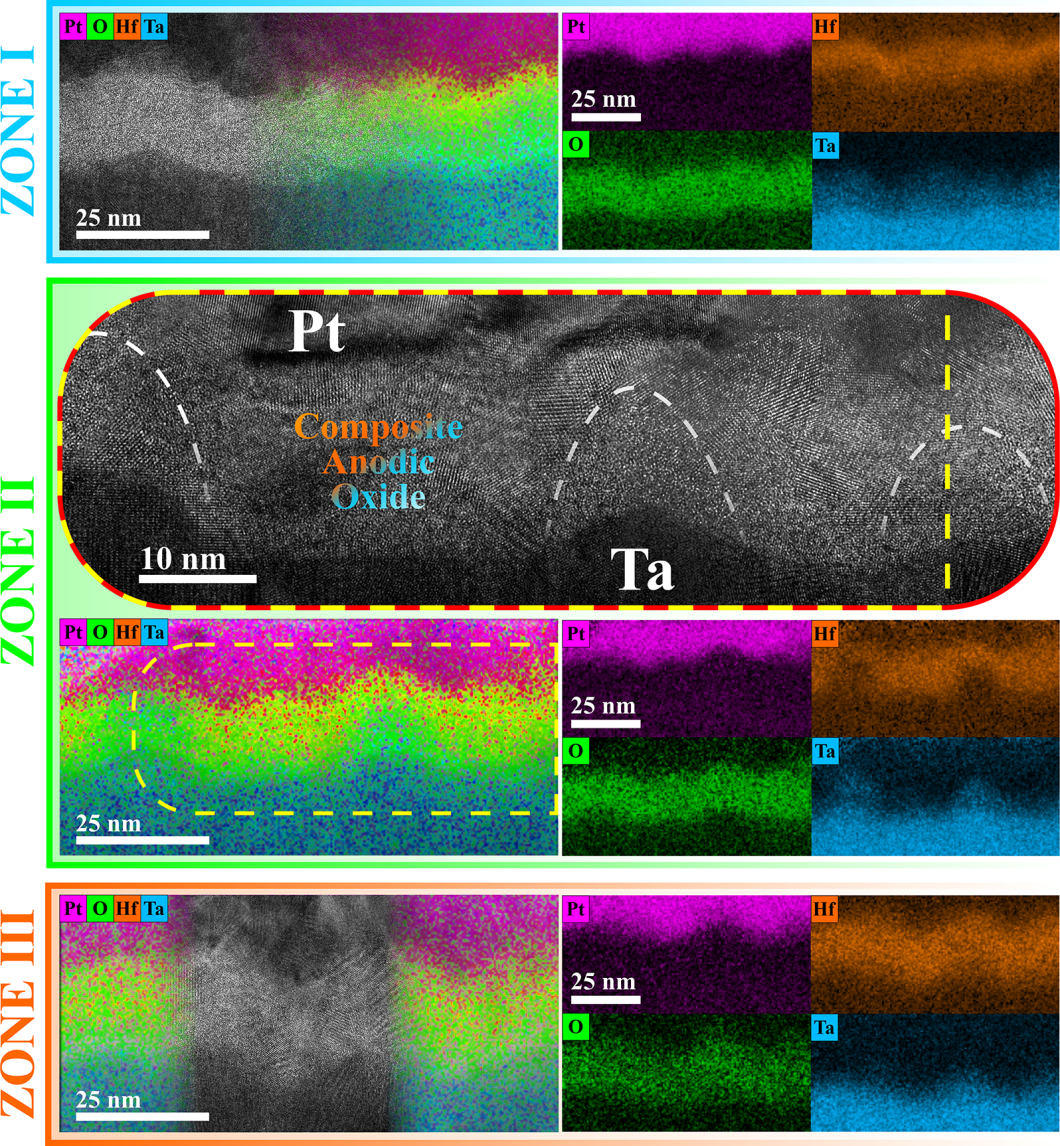
HRTEM images of memristors representative for zones I–III with the correspondent elemental EDX maps. The maps were reconstructed by using the L series of characteristic X-ray spectra for Pt, Hf, and Ta and the K series for O. Approximate position of the Ta_2_O_5_ “fingers” depicted in the HRTEM image of the zone II memristor structure with white dashed lines.

**Figure 4:**
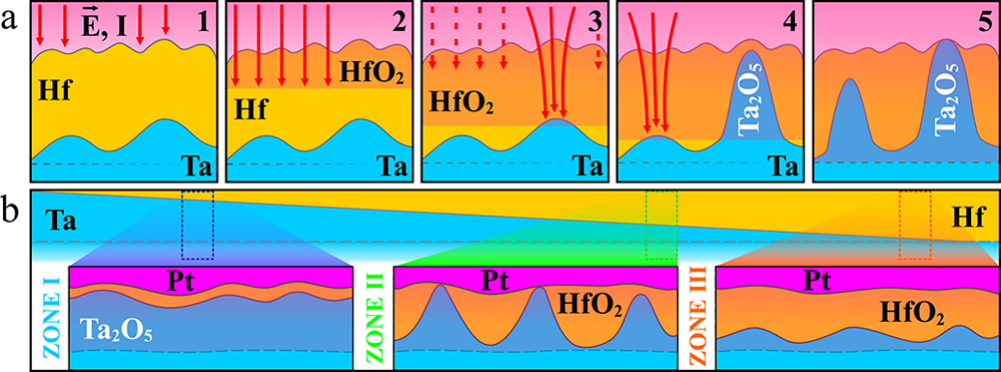
(a) Anodic oxidation steps in the Hf/Ta system leading to *in situ* nanostructuring and (b) layer thickness effects suggesting the existence of an optimum Hf/Ta thickness ratio where the best memristive performance can be found.

The ionic transport number for Hf (relatively to O) is below 0.05, suggesting that Hf cations cannot actually migrate toward the metal/electrolyte interface.^28^ Instead, O anions migrate inward against the electric field direction leading to HfO_2_ formation at the metal/electrolyte interface. For this reason, the oxidation of Hf leads to a negligible oxide growth inside the electrolyte, as suggested in Figure 4a by an invariable position of the oxide/electrolyte interface. Additionally, the Pilling–Bedworth ratio is higher for Ta.^28^ Because Ta_2_O_5_ is less resistive, a higher ratio will allow “fingers” to grow faster all the way through HfO_2_. The higher transport number of Ta (reported to Hf) may also benefit Ta oxide “fingers” formation. Such nanocolumns embedded in the Ta/Al system were reported as mainly Ta_2_O_5_, but also suboxides TaO_2_ and TaO_*x*_ (0.5 < *x* < 1) were found.^42^ Similarly, the structure of the oxide specific to Ta_2_O_5_ could also assist the “fingers” formation.^32^ This nanostructuring and the resulting boundaries between both oxides along these “fingers” (see Figure 3) may play a role in the memristive behavior of the entire composite oxide. Because of the high field oxide growth, O ions and vacancies can agglomerate along these boundaries, possibly facilitating CFs formation and deletion. The maintenance of a distinct interface between oxides is more probable between amorphous and crystalline ones, such as Ta_2_O_5_ and HfO_2_.

As reported by Pringle,^28^ any thickness variation of the superimposed metal will affect the nanostructure growth and shape peculiarities. This is suggested in Figure 4b for the Hf/Ta system superimposed as oppositely directed wedges. On the one hand, the excess of Ta (zone I) results in the formation of small oxide columns, since Hf will be fully anodized providing a small space for Ta_2_O_5_ “fingers” to reach the surface. Because the Ta_2_O_5_ growth mechanism relies on the outward migration of cations with a higher transport number (0.24 for Ta),^28,43^ the “finger” formation is justified even for thin Hf superimposed on Ta. However, electrolyte selection plays a crucial role in the switching behavior of Ta memristors. It was proven that incorporation of electrolyte species (such as P) may promote CFs pinning but was not observed when using CB.^24^ Thus, in the Ta-rich zone I the memristive behavior is poor. On the other hand, the excess of Hf superimposed on Ta (zone III) results in a too thick HfO_2_ layer, and the Ta source will be depleted before any of the growing Ta_2_O_5_ will have a chance to reach the surface. Therefore, no CFs can be formed and very high voltage values should be applied for electroforming. Moreover, both Hf and Ta are simultaneously consuming O during oxide formation, leading to randomization of O vacancies in the composite oxide, which in turn affects switching reliability.^16^ In zone II the nanostructuring and oxide mixing is the strongest, clearly affecting the memristive behavior.

Consequentially, it may be concluded that the CFs positioning is predefined by the development of Ta_2_O_5_ columnar structures grown during the anodization process. It is also possible that few CFs may be found in parallel, according to TEM observations, showing more than one Ta_2_O_5_ “finger”. Previous studies on pure Hf anodic memristors have confirmed concurrent competing CFs formation.^16^ Thus, one could assume that the switching mechanism can be conditioned by the formation of oxides with such structures. The selected Hf/Ta ratio corresponding to those devices produced in zone II could be an excellent choice for improved memristors fabrication. Previous studies confirmed that a sudden diffusion of O vacancies into the CF ruptured region during the switching repetition may decrease the HRS values and cause device failure.^41^ Hence, the random nature of CFs growth may increase the probability of such detrimental events and thus must be avoided. Controlled O vacancies generation is a critical factor in switching uniformity and reproducibility.^41^ Therefore, oxide “fingers” formation is a promising electrochemical approach toward defect-engineered memristors. Further investigation of the composite oxide formation, particularly in the Hf/Ta superimposed system, is topical. Until now, such systems were not recognized in the literature for the ReRAM applications. This is highly promising since both memory and electrical characteristics are improved by the forming-free nature of the memristors with CFs mediated by oxide nanostructuring.

## References

[ref1] Romero F. J.; Toral-Lopez A.; Ohata A.; Morales D. P.; Ruiz F. G.; Godoy A.; Rodriguez N. Laser-Fabricated Reduced Graphene Oxide Memristors. Nanomaterials 2019, 9 (6), 1–13. 10.3390/nano9060897. PMC6630327 31248215

[ref2] Carstens N.; Vahl A.; Gronenberg O.; Strunskus T.; Kienle L.; Faupel F.; Hassanien A. Enhancing Reliability of Studies on Single Filament Memristive Switching via an Unconventional Cafm Approach. Nanomaterials 2021, 11 (2), 1–16. 10.3390/nano11020265. PMC7909531 33498494

[ref3] Sokolov A. S.; Abbas H.; Abbas Y.; Choi C. Towards Engineering in Memristors for Emerging Memory and Neuromorphic Computing: A Review. J. Semicond. 2021, 42 (1), 013101 10.1088/1674-4926/42/1/013101.

[ref4] Rabbani P.; Dehghani R.; Shahpari N. A Multilevel Memristor-CMOS Memory Cell as a ReRAM. Microelectron. J. 2015, 46 (12), 1283–1290. 10.1016/j.mejo.2015.10.006.

[ref5] Gale E. TiO_2_-Based Memristors and ReRAM: Materials, Mechanisms and Models (a Review). Semicond. Sci. Technol. 2014, 29 (10), 1–29. 10.1088/0268-1242/29/10/104004.

[ref6] Borghetti J.; Snider G. S.; Kuekes P. J.; Yang J. J.; Stewart D. R.; Williams R. S. Memristive Switches Enable Stateful Logic Operations via Material Implication. Nature 2010, 464 (7290), 873–876. 10.1038/nature08940. 20376145

[ref7] Xia Q.; Robinett W.; Cumbie M. W.; Banerjee N.; Cardinali T. J.; Yang J. J.; Wu W.; Li X.; Tong W. M.; Strukov D. B.; et al. Memristor-CMOS Hybrid Integrated Circuits for Reconfigurable Logic. Nano Lett. 2009, 9 (10), 3640–3645. 10.1021/nl901874j. 19722537

[ref8] Yin L.; Cheng R.; Wang Z.; Wang F.; Sendeku M. G.; Wen Y.; Zhan X.; He J. Two-Dimensional Unipolar Memristors with Logic and Memory Functions. Nano Lett. 2020, 20 (6), 4144–4152. 10.1021/acs.nanolett.0c00002. 32369375

[ref9] Kim S.; Du C.; Sheridan P.; Ma W.; Choi S.; Lu W. D. Experimental Demonstration of a Second-Order Memristor and Its Ability to Biorealistically Implement Synaptic Plasticity. Nano Lett. 2015, 15 (3), 2203–2211. 10.1021/acs.nanolett.5b00697. 25710872

[ref10] Upadhyay N. K.; Jiang H.; Wang Z.; Asapu S.; Xia Q.; Joshua Yang J. Emerging Memory Devices for Neuromorphic Computing. Adv. Mater. Technol. 2019, 4 (4), 1800589 10.1002/admt.201800589.

[ref11] Abdul Hadi S.; Humood K. M.; Abi Jaoude M.; Abunahla H.; Shehhi H. F. Al; Mohammad B. Bipolar Cu/HfO2/P++ Si Memristors by Sol-Gel Spin Coating Method and Their Application to Environmental Sensing. Sci. Rep. 2019, 9 (1), 1–15. 10.1038/s41598-019-46443-x. 31292515 PMC6620357

[ref12] Wu Q.; Dang B.; Lu C.; Xu G.; Yang G.; Wang J.; Chuai X.; Lu N.; Geng D.; Wang H.; et al. Spike Encoding with Optic Sensory Neurons Enable a Pulse Coupled Neural Network for Ultraviolet Image Segmentation. Nano Lett. 2020, 20, 8015 10.1021/acs.nanolett.0c02892. 33063511

[ref13] Yalagala B. P.; Sahatiya P.; Kolli C. S. R.; Khandelwal S.; Mattela V.; Badhulika S. V2O5 Nanosheets for Flexible Memristors and Broadband Photodetectors. ACS Appl. Nano Mater. 2019, 2 (2), 937–947. 10.1021/acsanm.8b02233.

[ref14] Ielmini D. Resistive Switching Memories Based on Metal Oxides: Mechanisms, Reliability and Scaling. Semicond. Sci. Technol. 2016, 31 (6), 1–25. 10.1088/0268-1242/31/6/063002.

[ref15] Chen S.; Noori S.; Villena M. A.; Shi Y.; Han T.; Zuo Y.; Pedeferri M. P.; Strukov D.; Lanza M.; Diamanti M. V. Memristive Electronic Synapses Made by Anodic Oxidation. Chem. Mater. 2019, 31 (20), 8394–8401. 10.1021/acs.chemmater.9b02245.

[ref16] Zrinski I.; Mardare C. C.; Jinga L. I.; Kollender J. P.; Socol G.; Minenkov A.; Hassel A. W.; Mardare A. I. Electrolyte-dependent Modification of Resistive Switching in Anodic Hafnia. Nanomaterials 2021, 11 (3), 1–18. 10.3390/nano11030666. PMC8001223 33800460

[ref17] Zaffora A.; Cho D. Y.; Lee K. S.; Di Quarto F.; Waser R.; Santamaria M.; Valov I. Electrochemical Tantalum Oxide for Resistive Switching Memories. Adv. Mater. 2017, 29 (43), 1–6. 10.1002/adma.201703357. 28984996

[ref18] Kim G. S.; Song H.; Lee Y. K.; Kim J. H.; Kim W.; Park T. H.; Kim H. J.; Min Kim K.; Hwang C. S. Defect-Engineered Electroforming-Free Analog HfOx Memristor and Its Application to the Neural Network. ACS Appl. Mater. Interfaces 2019, 11 (50), 47063–47072. 10.1021/acsami.9b16499. 31741373

[ref19] Kim K. M.; Choi B. J.; Hwang C. S. Localized Switching Mechanism in Resistive Switching of Atomic-Layer-Deposited Ti O2 Thin Films. Appl. Phys. Lett. 2007, 90 (24), 242906 10.1063/1.2748312.

[ref20] Spiga S.; Driussi F.; Congedo G.; Wiemer C.; Lamperti A.; Cianci E. Sub-1 Nm Equivalent Oxide Thickness Al-HfO2 Trapping Layer with Excellent Thermal Stability and Retention for Nonvolatile Memory. ACS Appl. Nano Mater. 2018, 1 (9), 4633–4641. 10.1021/acsanm.8b00918.

[ref21] Xue Q.; Wang Y. C.; Wei X. H. Synaptic Plasticity of Room-Temperature Fabricated Amorphous MoO x Film Based Memristor. Appl. Surf. Sci. 2019, 479, 469–474. 10.1016/j.apsusc.2019.02.092.

[ref22] Zaffora A.; Di Quarto F.; Habazaki H.; Valov I.; Santamaria M. Electrochemically Prepared Oxides for Resistive Switching Memories. Faraday Discuss. 2019, 213, 165–181. 10.1039/C8FD00112J. 30357186

[ref23] Zaffora A.; Franco F. Di; Quarto F. Di; Macaluso R.; Mosca M.; Habazaki H.; Santamaria M. The Effect of Nb Incorporation on the Electronic Properties of Anodic HfO 2. ECS J. Solid State Sci. Technol. 2017, 6 (4), N25–N31. 10.1149/2.0121704jss.

[ref24] Zrinski I.; Minenkov A.; Mardare C. C.; Kollender J. P.; Lone S. A.; Hassel A. W.; Mardare A. I. Influence of Electrolyte Selection on Performance of Tantalum Anodic Oxide Memristors. Appl. Surf. Sci. 2021, 565 (July), 150608 10.1016/j.apsusc.2021.150608.

[ref25] Lu J.; Kuo Y. Hafnium-Doped Tantalum Oxide High-k Dielectrics with Sub-2 Nm Equivalent Oxide Thickness. Appl. Phys. Lett. 2005, 87 (23), 1–3. 10.1063/1.2140482.

[ref26] Lu J.; Kuo Y.; Tewg J.-Y. Hafnium-Doped Tantalum Oxide High-k Gate Dielectrics. J. Electrochem. Soc. 2006, 153 (5), G410 10.1149/1.2180647.

[ref27] Rigo S.; Siejka J. Long Range Migration of Niobium during the Anodic Oxidation of Tantalum on Niobium and Aluminum on Niobium Duplex Layers. Solid State Commun. 1974, 15 (2), 259–264. 10.1016/0038-1098(74)90753-4.

[ref28] Pringle J. P. S. The Anodic Oxidation of Superimposed Metallic Layers: Theory. Electrochim. Acta 1980, 25 (11), 1423–1437. 10.1016/0013-4686(80)87157-X.

[ref29] Perrière J.; Rigo S.; Siejka J. Investigation of Cation-Transport Processes during Anodic Oxidation of Duplex Layers of Tantalum on Niobium by the Use of Rutherford Backscattering and Nuclear Microanalysis. J. Electrochem. Soc. 1978, 125 (9), 1549–1557. 10.1149/1.2131713.

[ref30] Mozalev A.; Gorokh G.; Sakairi M.; Takahashi H. The Growth and Electrical Transport Properties of Self-Organized Metal/Oxide Nanostructures Formed by Anodizing Ta-Al Thin-Film Bilayers. J. Mater. Sci. 2005, 40 (24), 6399–6407. 10.1007/s10853-005-1620-9.

[ref31] Mozalev A.; Vázquez R. M.; Bittencourt C.; Cossement D.; Gispert-Guirado F.; Llobet E.; Habazaki H. Formation-Structure-Properties of Niobium-Oxide Nanocolumn Arrays via Self-Organized Anodization of Sputter-Deposited Aluminum-on-Niobium Layers. J. Mater. Chem. C 2014, 2 (24), 4847–4860. 10.1039/c4tc00349g.

[ref32] Pringle J. P. S. The Anodic Oxidation of Superimposed Niobium and Tantalum Layers: Theory. Electrochim. Acta 1980, 25 (11), 1403–1421. 10.1016/0013-4686(80)87156-8.

[ref33] Mardare A. I.; Ludwig A.; Savan A.; Wieck A. D.; Hassel A. W. Combinatorial Investigation of Hf-Ta Thin Films and Their Anodic Oxides. Electrochim. Acta 2010, 55 (27), 7884–7891. 10.1016/j.electacta.2010.03.066.

[ref34] Zrinski I.; Mardare C. C.; Jinga L.-I.; Kollender J. P.; Socol G.; Hassel A. W.; Mardare A. I. Phosphate Incorporation in Anodic Hafnium Oxide Memristors. Appl. Surf. Sci. 2021, 548, 149093 10.1016/j.apsusc.2021.149093.

[ref35] Sodium Phosphate:

[ref36] Goux L.; Lisoni J. G.; Jurczak M.; Wouters D. J.; Courtade L.; Muller C. Coexistence of the Bipolar and Unipolar Resistive-Switching Modes in NiO Cells Made by Thermal Oxidation of Ni Layers. J. Appl. Phys. 2010, 107 (2), 024512 10.1063/1.3275426.

[ref37] Gao B.; Kang J. F.; Chen Y. S.; Zhang F. F.; Chen B.; Huang P.; Liu L. F.; Liu X. Y.; Wang Y. Y.; Tran X. A.; et al. Oxide-Based RRAM: Unified Microscopic Principle for Both Unipolar and Bipolar Switching. Technol. Dig. - Int. Electron Devices Meet. IEDM 2011, 417–420. 10.1109/IEDM.2011.6131573.

[ref38] Mohammad B.; Jaoude M. A.; Kumar V.; Al Homouz D. M.; Nahla H. A.; Al-Qutayri M.; Christoforou N. State of the Art of Metal Oxide Memristor Devices. Nanotechnol. Rev. 2016, 5 (3), 311–329. 10.1515/ntrev-2015-0029.

[ref39] Chen L.; Li C.; Huang T.; Hu X.; Chen Y. The Bipolar and Unipolar Reversible Behavior on the Forgetting Memristor Model. Neurocomputing 2016, 171, 1637–1643. 10.1016/j.neucom.2015.06.067.

[ref40] Wang W.; Zhang B.; Zhao H. Forming-Free Bipolar and Unipolar Resistive Switching Behaviors with Low Operating Voltage in Ag/Ti/CeO_2_/Pt Devices. Results Phys. 2020, 16, 103001 10.1016/j.rinp.2020.103001.

[ref41] Kim G. S.; Park T. H.; Kim H. J.; Ha T. J.; Park W. Y.; Kim S. G.; Hwang C. S. Investigation of the Retention Performance of an Ultra-Thin HfO2 Resistance Switching Layer in an Integrated Memory Device. J. Appl. Phys. 2018, 124 (2), 024102 10.1063/1.5033967.

[ref42] Mozalev A.; Smith A. J.; Borodin S.; Plihauka A.; Hassel A. W.; Sakairi M.; Takahashi H. Growth of Multioxide Planar Film with the Nanoscale Inner Structure via Anodizing Al/Ta Layers on Si. Electrochim. Acta 2009, 54 (3), 935–945. 10.1016/j.electacta.2008.08.030.

[ref43] Sloppy J. D.; Lu Z.; Dickey E. C.; MacDonald D. D. Growth Mechanism of Anodic Tantalum Pentoxide Formed in Phosphoric Acid. Electrochim. Acta 2013, 87, 82–91. 10.1016/j.electacta.2012.08.014.

